# Novel drug combination nanoparticles exhibit enhanced plasma exposure and dose-responsive effects on eliminating breast cancer lung metastasis

**DOI:** 10.1371/journal.pone.0228557

**Published:** 2020-03-06

**Authors:** Qingxin Mu, Jesse Yu, James I. Griffin, Yan Wu, Linxi Zhu, Lisa A. McConnachie, Rodney J. Y. Ho

**Affiliations:** 1 Department of Pharmaceutics, University of Washington, Seattle, WA, United States of America; 2 Department of Bioengineering, University of Washington, Seattle, WA, United States of America; University of Colorado Denver Skaggs School of Pharmacy and Pharmaceutical Sciences, UNITED STATES

## Abstract

Early diagnosis along with new drugs targeted to cancer receptors and immunocheckpoints have improved breast cancer survival. However, full remission remains elusive for metastatic breast cancer due to dose-limiting toxicities of heavily used, highly potent drug combinations such as gemcitabine and paclitaxel. Therefore, novel strategies that lower the effective dose and improve safety margins could enhance the effect of these drug combinations. To this end, we developed and evaluated a novel drug combination of gemcitabine and paclitaxel (GT). Leveraging a simple and scalable drug-combination nanoparticle platform (DcNP), we successfully prepared an injectable GT combination in DcNP (GT DcNP). Compared to a Cremophor EL/ethanol assisted drug suspension in buffer (CrEL), GT DcNP exhibits about 56-fold and 8.6-fold increases in plasma drug exposure (area under the curve, AUC) and apparent half-life of gemcitabine respectively, and a 2.9-fold increase of AUC for paclitaxel. Using 4T1 as a syngeneic model for breast cancer metastasis, we found that a single GT (20/2 mg/kg) dose in DcNP nearly eliminated colonization in the lungs. This effect was not achievable by a CrEL drug combination at a 5-fold higher dose (i.e., 100/10 mg/kg GT). A dose-response study indicates that GT DcNP provided a therapeutic index of ~15.8. Collectively, these data suggest that GT DcNP could be effective against advancing metastatic breast cancer with a margin of safety. As the DcNP formulation is intentionally designed to be simple, scalable, and long-acting, it may be suitable for clinical development to find effective treatment against metastatic breast cancer.

## Introduction

Breast cancer is a leading cause of death in women in the US and worldwide. Estimates suggest that in 2019 over 270,000 people will be newly diagnosed and that 42,000 people will die of the disease in the US alone [1]. The NIH, the Congressionally Directed Medical Research Program (CDMRP), and other foundations have made significant progress through ~ $1 billion in annual research investment. However, a cure for breast cancer remains elusive. Early diagnosis, resection of breast cancer nodules, and receptor targeted therapeutics (that inhibit human epidermal growth factor and hormone receptors) are effective at extending survival rates. However, many cancers still progress to the metastatic stage due to drug resistance and genetic mutation/evolution. Treatment options for these metastatic breast cancer patients are limited and outcomes are dismal. Even with current best agents—including drug-combinations and multiple cycle chemotherapy—treatments provide about 27% five-year survival [1]. Patients at the metastatic stage exhibit cancer cells spread to highly perfused organs and local lymph nodes, detectable as colonies and nodules [2]. Physiological mechanisms and the time-course of cancer cells metastasizing into lymph nodes and tissues are not fully understood. This gap has prevented the development of treatment interventions, specifically those targeted to these sites early in the course of advancing cancer.

Two recent reports from separate laboratories have provided time-and-spatial insight into the metastatic spread of breast cancer cells from primary sites (nodes and mammary gland) into blood (becoming apparent in the lungs as nodules) (Brown et al, Pereira et al, Science, 359, 2018) [3, 4]. The two independent studies using 4T1 metastatic mouse tumors as models suggest that either removal of primary tumor or introducing a small number of cells in lymph node cortex (within the sinuses) would invariably lead to their appearance (through invasion into blood) in the lungs as nodules or colonies of breast cancer cells. The studies suggest that cancer cells rapidly proliferate in blood and migrate into the lungs to form colonies detectable as nodules [3]. The time-course and spatial 4T1 tumor spread data thus suggests that early systemic intervention with highly active chemotherapeutic or targeted agents responsive to metastatic cells could enhance response rate in metastatic breast cancer and delay the rate of disease progression.

According to NCCN (National Comprehensive Cancer Network) guidelines in the US, patients with newly diagnosed or recurrent breast cancer are treated with surgery if applicable prior to multiple cycles of adjuvant therapy [5]. Metastatic breast cancer patients are often treated with intensive chemotherapeutic drug combinations targeted to topoisomerase or DNA synthesis plus microtubules, such as doxorubicin and paclitaxel, gemcitabine and paclitaxel (GT), capecitabine and docetaxel, or capecitabine and ixabepilone. These combination chemotherapies, while more effective than monotherapies, often exhibit dose-limiting toxicities, and intolerabilities prevent patients from completing their treatment cycles. For example, gemcitabine (1250 mg/m^2^ IV day 1, day 8) and paclitaxel (175 mg/m^2^ IV d1) combinations are reported to provide 41.4% response rate compared to paclitaxel alone (26.2%) [6]. Median survival of this combination as a first-line treatment was 18.6 months versus 15.8 months on paclitaxel only. In another study in patients who failed neo-adjuvant anthracycline-based chemotherapy, the same dose regimen produces a 50% objective response rate in the 12-month study [7]. However, significant side effects such as neutropenia, leukopenia, and poor tolerability were reported for these combination therapies.

Collectively, if one can co-deliver an effective drug combination to advancing metastatic breast cancer cells, a lower overall dose would be needed to overcome dose-limiting toxicities.

In developing long-acting (LA) and combination anti-retroviral (drug) therapeutics or cART, our laboratory has discovered a simple, scalable technology that enables the incorporation of 2–3 HIV drugs that are water insoluble such as lopinavir, ritonavir, atazanavir, and efavirenz together with water soluble tenofovir and lamivudine [8–11]. This drug-combination nanoparticle (DcNP) technology has proven to be a platform that allows high loading of both hydrophobic and hydrophilic HIV drugs. With at least 4 different sets of cART tested in non-human primates, DcNP enhances lymphoid tissue and cell drug accumulation while exhibiting long-acting cellular exposure [8–11]. With this novel enabling technology, we have investigated whether gemcitabine (G, soluble) and paclitaxel (T, insoluble) can be assembled into a drug-combination particle able to enhance pharmacokinetics and also inhibit the growth of metastatic breast cancer. To this end, we leverage knowledge gained from recent studies using the advancing 4T1 cells in the lungs as a surrogate marker of disease progression [3, 4]. Our results suggest that a single dose of DcNP formulated GT combination (20/2 mg/kg GT in DcNP) could reduce 4T1 to nearly non-detectable levels by day 14, while there was little to no effect on 4T1 with equivalent CrEL drug dosing.

## Materials and methods

### Reagents and cell line

1,2-Distearoyl-sn-glycero-3-phosphocholine (DSPC) and N-(carbonylmethoxypolyethyleneglycol with MW = 2000)-1,2-distearoyl-sn-glycero-3-phosphoethanolamine, sodium salt (DSPE-mPEG_2000_) (GMP grade) were purchased from Corden Pharma (Liestal, Switzerland). Paclitaxel (>99.5%), gemcitabine free form (>99%), and gemcitabine hydrochloride (>99%) were purchased from LC Laboratories (Woburn, MA). All other chemicals and reagents were analytical grade or higher. 4T1 cell line transfected and verified to express luciferase and green fluorescence protein (GFP) (referred to as 4T1-luc) was kindly provided by Stanley Riddell laboratory [12], Fred Hutchinson Cancer Research Center.

### Formulation and characterization of GT DcNP

DcNP composed of DSPC and DSPE-mPEG_2000_ as lipid excipients, paclitaxel, and gemcitabine (90:10:2.5:80 molar ratio) were prepared aseptically as follows: Lipid excipients and drugs were solubilized together in ethanol at 60°C. Ethanol was removed by controlled solvent evaporation at 60°C, followed by vacuum desiccation to remove residual solvent. The dry film was rehydrated to 100 mM lipids in 0.45% NaCl with 20 mM NaHCO_3_ buffer at 60°C for 2 h. Particle size was reduced at ~40°C using a bath sonicator (Avanti Polar Lipids, Inc. Alabaster, AL) (5 min on, 5 min off, 3 cycles). GT DcNP formulations were stored at room temperature for further use. Particle size was determined by a NICOMP 380 ZLS (Particle Sizing Systems, Santa Barbara, CA). Drug extraction with acetonitrile followed by HPLC were used to quantify drugs in formulations. Drug association was measured by dialysis (6–8 kDa) of DcNP against 0.9% NaCl 20 mM NaHCO_3_ buffer for 4 h and quantification by HPLC.

### Preparation of GT CrEL drug combination

Paclitaxel was dissolved in ethanol (20 mg/mL) and diluted with an equal volume of Cremophor EL (Sigma-Aldrich, St. Louis, MO). The solution was then diluted 10× with a premade PBS solution of gemcitabine (hydrochloride salt) (12.65 mg/mL). Final concentrations of drugs were 10/1 mg/mL GT. CrEL drug suspensions were used within the same day of preparation due to instability.

### Pharmacokinetic study

All animal studies were conducted in accordance with University of Washington Institute of Animal Care and Use Committee (IACUC) approved protocols (protocol number 2372–06). The UW IACUC has specifically approved this study. Isoflurane was used for anesthesia during live animal imaging. 5–6 week-old female BALB/c mice were purchased from The Jackson Laboratory (Bar Harbor, Maine) and housed in an animal research facility for at least one week before use.

Mice were administered with either a CrEL drug combination or GT DcNP intravenously with doses of 50/5 mg/kg GT. Blood was collected through retro-orbital bleeding at 5, 60, 120, 360 min for CrEL GT and 5, 60, 120, 360, 1440, 4320 min (72 h) for GT DcNP. Each group had three animals and each animal was bled once only. Retro-orbital blood collection in this study was a terminal procedure and animals were under anesthesia at the time of bleeding. After blood collection, mice were euthanized by CO_2_ overdose followed by cervical dislocation as the secondary method of euthanasia. Drugs in plasma were extracted and analyzed by LC-MS/MS as described below.

#### Drug extraction

A liquid-liquid extraction was used to extract drugs from plasma or tissue homogenates. 50 μL of sample were transferred into 1.5 mL tubes with or without dilution by blank matrix to an appropriate concentration range. Samples were spiked by internal standards (see below) followed by the addition of acetonitrile. Samples were then vortexed and centrifuged at 4°C for 15 minutes at 14000 rpm. The supernatant was then removed and dried under nitrogen at 40°C. The dried samples were reconstituted in 20% methanol and 80% water in 50 μL.

#### Quantification of drugs by LC-MS/MS

Drugs were quantified by a Shimadzu HPLC system coupled to a 3200 QTRAP mass spectrometer (Applied Biosystems, Grand Island, NY). The HPLC system consisted of two Shimadzu LC-20A pumps, a DGU-20A5 degasser, and a Shimadzu SIL-20AC HT autosampler. The mass spectrometer was equipped with an electrospray ionization (ESI) TurboIonSpray source. The system was operated with Analyst software, version 1.5.2 (ABSciex, Framingham, MA).

Chromatographic separation of drugs was achieved using a Synergi column (100 × 2.0 mm; 4- μm particle size) with an inline C8 guard column (4.0 × 2.0 mm) (Phenomenex, Torrance, CA). An ammonium acetate buffer/reagent alcohol gradient was used to separate components. Analytes were monitored using multiple-reaction monitoring for positive ions. The following ion transitions were monitored: gemcitabine, m/z 264.066→112.000; paclitaxel, m/z 854.266→286.200; a stable labeled isotope (C_8_^13^CH_12_ClF_2_N^15^N_2_O_4_) (m/z 267.067→115.100) was used as an internal standard for gemcitabine; docetaxel (m/z 830.312→549.3) was used as an internal standard for paclitaxel.

### 4T1 cell inoculation

Six-week-old, female BALB/c mice were used in this study. 4T1 cells were transfected with luciferase and green fluorescence protein (GFP) (4T1-luc); thus 4T1 growth could be monitored based on that bioluminescence [13]. 4T1-luc (0.5, 1 or 2 × 10^5^ cells) suspended in a 100 μL ice-cold HBSS suspension was intravenously inoculated through mouse tail veins. Mice were monitored for a two-week period. Bioluminescence of 4T1-luc from living mice was examined by a XENOGEN IVIS 200 imaging system (PerkinElmer, Inc. Waltham, MA). Mice received 150 mg/kg D-luciferin through intraperitoneal injections 10~15 min before imaging. The bioluminescence imaging parameters for living mice were set as follows: field of view, 12; excitation filter, closed; emission filter, open; exposure time, 120 sec; binning factor, 4; f/stop, 2. Total 4T1-luc bioluminescence emission from living mice was integrated using Live Image software (PerkinElmer, Waltham, MA).

### Effects of CrEL drug combinations and DcNP on metastatic breast cancer colony formation in the lung

Six-week-old, female BALB/c mice were inoculated with 2 × 10^5^ 4T1-luc cells IV in 100 μL HBSS on day 0. Three hours later, mice were given a single administration of saline, a CrEL drug combination, or GT DcNP through IV injections (n = 8–15). The GT doses were 50/5 mg/kg for CrEL and DcNP formulations. On day 14, mice were euthanized immediately after live imaging and lungs were collected and placed in 12-well plates to quantify luminescence images. The images were acquired by a Xenogen IVIS-200. The bioluminescence imaging parameters for living mice were set as follows: field of view, 24; excitation filter, closed; emission filter, open; exposure time, 180 sec; binning factor, 4; f/stop, 2. The imaging parameters for lungs were set as follows: field of view, 10; excitation filter, closed; emission filter, open; exposure time, 30 sec; binning factor, 4; f/stop, 2. Bioluminescence intensity from living mice and lungs was integrated using Live Image software. Mouse lung tissue was fixed in formalin and stored in 70% EtOH before being embedded in paraffin blocks. GFP staining of thin sections (5 μm) was carried out by UW histology and imaging core.

### Dose dependence of GT DcNP on 4T1 metastases

Six-week-old, female BALB/c mice were inoculated with 2 × 10^5^ 4T1-luc cells in 100 μL HBSS through IV injections on day 0. Three hours later, mice received a single administration of saline or a different dosage of GT DcNP through IV injections (n = 8–15). The dosages for DcNP were 0.125/0.0125, 1.25/0.125, 5/0.5, 10/1, and 20/2 mg/kg GT, respectively. Mouse behavior and overall health conditions were observed on a daily basis and body weight was measured every 2 days. On day 14, bioluminescence from living mice was examined with the IVIS imaging system as described above. Lung metastasis was detected by isolating lungs and imaging with IVIS as described above. After euthanasia, all organs were collected and visually examined for apparent toxicity.

### Statistical analysis

Data were presented as mean ± standard error of the mean (SEM). The number of mice in all groups ranges from 8 to 15. Students’ t-tests were performed for two groups, and the statistical significance was evaluated using one-way ANOVA for multiple groups. A P-value of <0.05 was considered statistically significant. Statistical analyses were performed using GraphPad Prism (Version 7.0).

## Results

### Development characterization of gemcitabine and paclitaxel together in an injectable DcNP formulation

We first determined whether a water soluble gemcitabine (LogP = -1.5) and insoluble paclitaxel (LogP = 3) could be integrated into a drug combination particle in suspension presented as an injectable dosage form. Employing a composition of lipid excipients previously reported to enable stabilization and formulation of 3-to-4 HIV drugs that exhibit disparate hydrophobicity in each set of DcNP [9], we tested the GT combination. We found that at a fixed G:T 32:1 (m/m; equals to 10:1 w/w) and lipid excipients–DSPC, DSPE-mPEG_2000_ (9:1 m/m), a stable and scalable DcNP with approximately 60 nm diameter can be made. We have tested at least 4 batches of DcNP preparation and verified that this process is reproducible, that DcNP products are stable, and that they can be scaled-up for the *in vivo* studies described. As the resulting GT DcNP product is less than 200 nm in diameter and stable in suspension (for at least 3 months and amenable for sterilization by 0.2 μm filtration), it is suitable for IV administration. Since the current clinical dose for GT is approximately 10:1 (w/w) (gemcitabine 1000~1250 mg/m^2^, paclitaxel 80~175 mg/m^2^), we used the DcNP with a similar drug ratio for the studies in mice described below.

### Enhanced plasma gemcitabine and paclitaxel exposure when presented in DcNP dosage form

We subsequently determined the effect of GT DcNP on a plasma drug-concentration time course of co-formulated GT as injectable dosage form. Compared to a CrEL drug combination counterpart, the two drugs in the GT DcNP formulation greatly improve the total plasma drug exposure of GT at an equivalent dose. After a 50/5 (GT) mg/kg IV dose, gemcitabine in DcNP exhibits about 56-fold higher exposure (AUC) and 8.6-fold longer apparent half-life than an equivalent CrEL drug combination dosage in mice (Table 1). The dramatic increase in gemcitabine AUC per dose is reflected in both a small (~10%) increase in C_max_ and an ~8.7-fold increase in apparent half-life. At a 10-fold lower dose than gemcitabine, paclitaxel in the fixed dose combination (5 mg/kg) given in GT DcNP exhibited an ~21% decrease in C_max_ but a similar half-life (1.97 vs 1.81 h). Due to the higher persistence of paclitaxel in DcNP, the overall AUC enhancement is about 2.9 fold (Table 1). Collectively, these data indicate that a co-formulation of GT in DcNP can provide longer acting and higher GT exposure in mice compared to CrEL drug dosages.

**Table 1:** The effect of gemcitabine and paclitaxel presented in a drug combination nanoparticle platform (DcNP) dosage form on the select pharmacokinetic parameters of the two drugs, compared to a CrEL drug dosage control form*.

	Gemcitabine (G)		Paclitaxel (T)	
	CrEL	DcNP^a^	CrEL	DcNP
**C**_**max**_ **(**μ**g/mL)**	165.12^b^	181.4	17.7	13.9
**AUC**_**0→t**_ **(**μ**g min/mL)**	917.6	52063.7	149.29	588.75
**t**_**1/2apparent**_ **(h)**	1.60	13.72	1.81	1.97

*GT (50/5 mg/kg in 100 μl) in DcNP or CrEL drug dosage form was given intravenously to mice. Plasma drug concentrations were determined over 3 days. The plasma drug concentration time course was analyzed and the listed pharmacokinetic parameters are generated based on non-compartmental analysis (n = 3 composite sampling).

^a^CrEL drugs scaled to equivalent DcNP dosages.

^b^Geometric Mean (95% CI).

Abbreviations: C_max_, maximum plasma drug concentration; AUC_0→t_, area under the plasma drug concentration-time curve to experimental time point; t_1/2_, apparent terminal plasma drug half-life.

### Characterization of 4T1-luc in BALB/c mice as a syngeneic metastatic tumor establishment and nodule growth model for intervention studies

To determine whether enhanced GT exposure can translate into improvements in inhibition of metastatic tumor establishment and growth, we first evaluated if 4T1 inoculated intravenously into BALB/c mice could model hematogenous metastasis. 4T1 introduced into the blood have recently been shown to establish and grow in the lungs as nodules, and at a much faster rate than in the lymph nodes and the sinuses [3]. In addition, the 4T1 (labeled with luciferase for live tracking) are able to invade blood capillaries in the nodes and spread to lungs, which are detectable as 4T1 nodules [4]. Therefore, we first performed a titration study to find a dose of 4T1 cells that produces tumor nodules in the lungs while not overburdening the mice with tumors. To do so, we employed a 4T1 cell line carrying luciferase marker (4T1-luc) [13]. The transfection of luciferase did not affect cell proliferation or migration [3]. After verifying luciferase expression by the 4T1-luc, these breast cancer cells were inoculated into the tail veins of BALB/c female mice. We studied a dose range between 50 to 200 × 10^3^ 4T1-luc cells in the inoculum per mouse). Bioluminescence (of 4T1-luc), body weight, and general behavior were monitored over two weeks. We found that bioluminescence signals increased exponentially from days 10 to 13 (from 0.5 to 3.5 × 10^5^ photo counts). Furthermore, the body weight of mice gradually declined (~10% from day 10 to day 13) at a higher inoculum dose in mice corresponding to the exponential increase in the lung bioluminescence intensity. We found, with 200 × 10^3^ cells, that about 20~30 tumor nodules and 2.0~3.0 × 10^5^ photon counts of bioluminescence could be detected in the mouse lungs—showing that colonies establish and grow over time in these tissues with acceptable weight and overall health for interventional studies. Thus, 200 × 10^3^ 4T1-luc cells in the inoculum was used for the following studies.

It is well known that animal studies are highly variable due to individual differences, batch variances, etc. To verify the reproducibility of this model, we repeated our study five times with a total of 21 mice and compared lung bioluminescence intensities using a 200 × 10^3^ cell inoculation number. Results indicate that the model is highly reproducible and reliable with 100% tumor uptake and no significant difference between the mean bioluminescence (*p* = 0.0681 by one-way ANOVA). The rapid and aggressive 4T1 tumor growth at this dose has limited our ability to keep untreated mice for up to14 days. The effectiveness studies in following sections were determined using a 200 × 10^3^ 4T1 cell inoculation while carrying saline controls for each set of experiments or replications.

### Effects of DcNP on gemcitabine and paclitaxel combinations for inhibiting 4T1 syngeneic mouse metastasis

**Fig 1:**
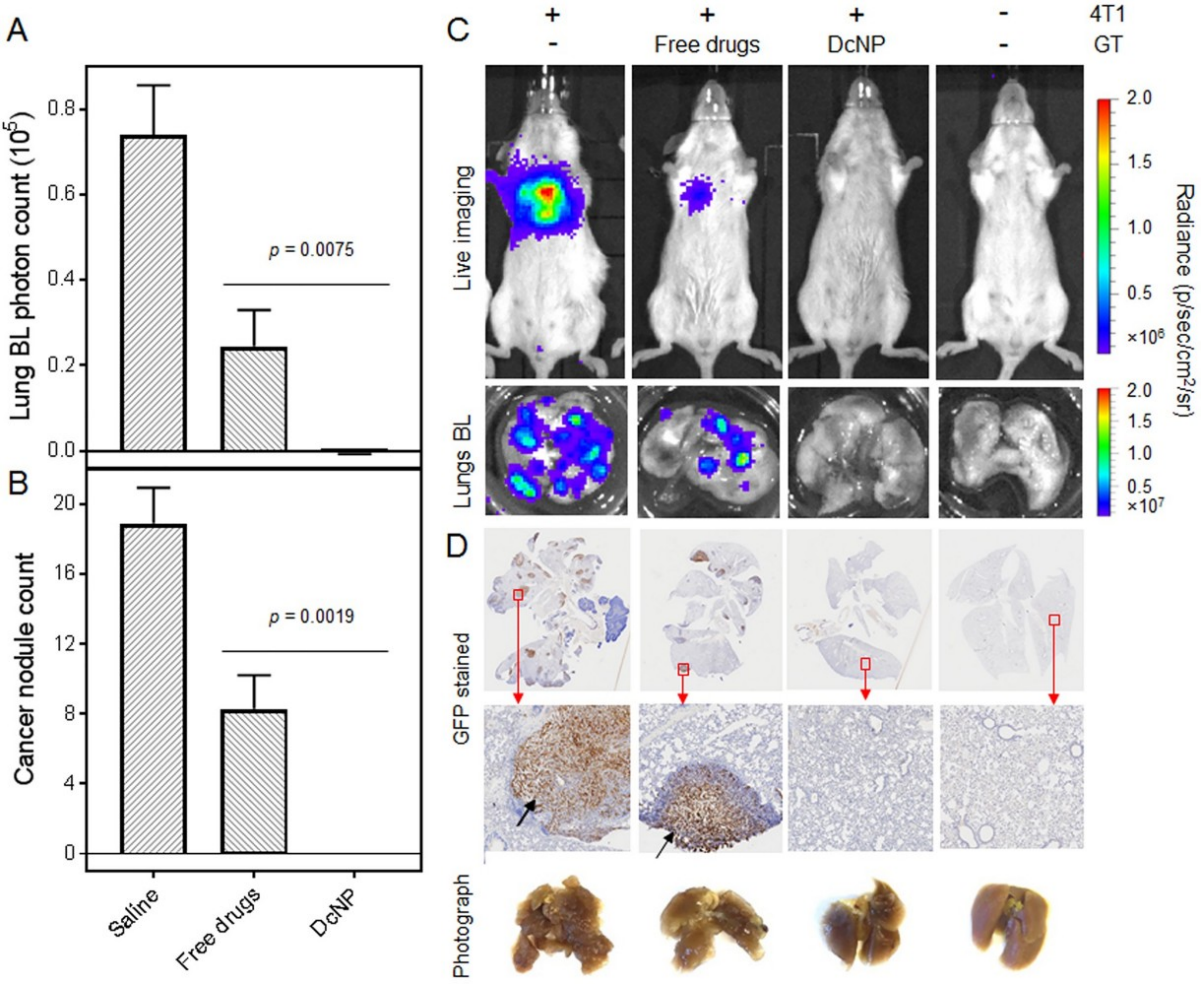
Effect of DcNP on gemcitabine and paclitaxel fixed-dose combination treatment on 4T1 metastatic tumor intensity and nodules in the lungs. Mice inoculated with 4T1-luc via tail vein were administered with a 50/5 mg/kg GT fixed dose combination in DcNP (test) or CrEL (control) formulation as a single bolus IV dose. On day 14, the total tumor growth was estimated based on luciferase activity detected as total bioluminescence (BL) intensity (A), and the cancer nodule count (B). Equivalent single IV doses of conventional formulation of the GT fixed dose combination was given to mice as a control. The values in panels (A) and (B) are expressed as mean ± SEM. *P* values were obtained from two-tailed t-tests with unequal variations. Experimental animal numbers in each group were 8–15. (C) Representative 4T1-luc luciferase mediated bioluminescence intensities in saline control, CrEL drug combination, DcNP treated mice, and healthy mice, as well as the lung nodules harvested from these mice. (D) Images of GFP (expressed by 4T1-luc) stained lung cross-sections from mice in conditions of (C), and photographs of fixed lung tissues. Top row, whole lung cross-sections; Bottom row, enlarged images from red boxes in top row. Black arrows indicate cancer nodules.

To determine the effects of enhanced GT exposure when presented in DcNP dosage form, we first used 50/5 mg/kg (GT) based on the current clinical (surface area converted to weight based) dose. Mice were first inoculated with 4T1 cells and given a single IV dose of GT either in CrEL or DcNP form. We intentionally chose identical GT doses (50/5 mg/kg) for the two formulations to evaluate DcNP effect on this treatment model (and without using dose compensations to match plasma drug exposures between the two formulations). The short interval between cell inoculation and GT administration (3h) was also purposefully designed, as the goals of this study were examining the clearance of advancing cancer cells in blood and eliminating formation of lung metastasis nodules. Tumor nodule formation was monitored over 14 days. As shown in Fig 1A and 1B, at day 14 mice treated with GT DcNP formulation completely inhibited 4T1 colonies in the lungs while the CrEL dosage form only inhibited 60~70%. The bioluminescence intensity data are verified with lung nodule counts and ex-vivo 4T1-luc-luminescence verification of the excised lungs (Fig 1C). However, we noticed a trend toward weight loss around day 4–6 in mice treated with 50/5 GT mg/kg or higher dose. These quantitative data indicate that a single dose of GT co-formulated in DcNP could completely inhibit the establishment and growth of 4T1 metastatic cancer in the lungs at a significantly higher rate than that provided by CrEL drug combinations in this syngeneic mouse model.

To further characterize lung metastasis and treatments at the microscopic level, lung sections were examined with GFP immunohistochemistry given GFP is expressed by 4T1-luc, and the microstructures were evaluated in comparison to controls (Fig 1D). First, we observed that the metastatic nodules in lungs often occur near blood vessels, indicating that 4T1-luc cells deposit in lungs following deposition in narrow vasculature. Second, we evaluated the lung tissues of 4T1 inoculated mice treated with placebo or drug combinations. As shown in Fig 1D (second rows of graphs), the cross-sections of lungs treated with CrEL dosage form and placebo exhibited a larger number of 4T1 cells positive to GFP while those from a GT DcNP single dose treatment did not. These data are consistent with bioluminescence measures in-life and ex-vivo of lung analysis where we found a high single IV dose GT DcNP treatment completely cleared 4T1 cancer nodules in the lungs.

### Dose-dependent tumor inhibitory and gross toxicity (weight loss) effect of GT combination in DcNP to estimate therapeutic index

**Fig 2:**
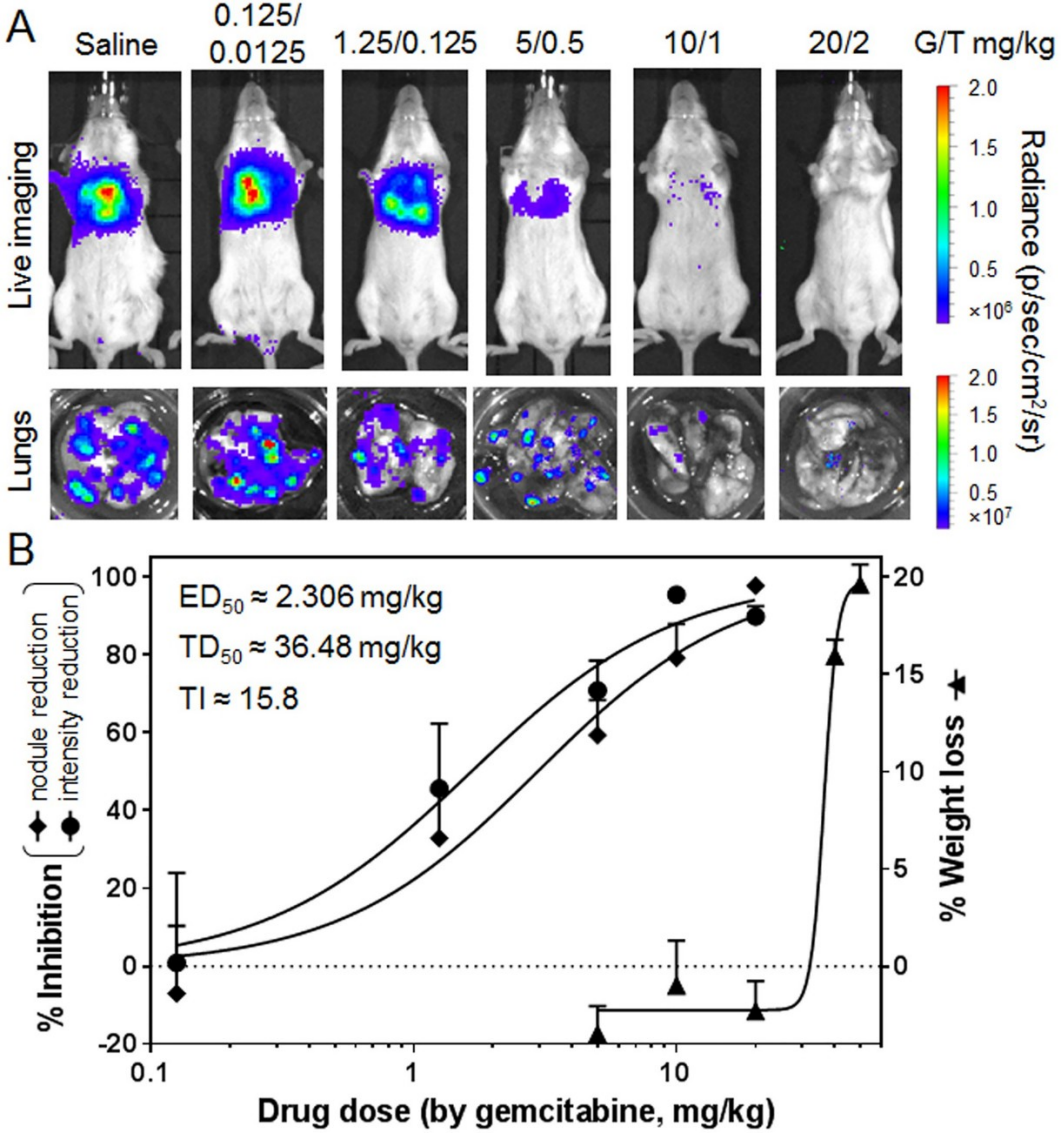
The dose-response of DcNP formulated gemcitabine-paclitaxel on inhibiting 4T1 lung metastasis; and bodyweight reduction. The 4T1-luc breast cancer cells were inoculated via tail-vein and the indicated dose (anchored on gemcitabine containing 1/10 weight equivalent of paclitaxel in DcNP formulation) were administered as a single dose IV administration. The 4T1 tumor growth (based on 4T1-luc luciferase dependent bioluminescence) and tumor nodule counts were expressed as therapeutic effects. The bodyweight loss at day 4 was used as an indicator of gross toxicity. (A) Representative 4T1-luc luciferase mediated bioluminescence intensities in saline and DcNP (with different GT doses) treated mice, as well as the lung nodules harvested from these mice. (B) Dose-responsive curves of metastasis inhibition were determined by bioluminescence integration and nodule count, as well as body weight loss with DcNP treatment. The values expressed are mean ± SEM. Experimental animal numbers in each group were 8–15. The curves were fitted in GraphPad Prism software (dose response-inhibition) to estimate ED_50_s and TD_50_s based on gemcitabine doses. The ED_50_ was averaged from two measures. The average therapeutic index (TI) is estimated based on the ratio of TD_50_-to-ED_50_ which is 15.8.

To confirm the initial results of the GT fixed-ratio combination in DcNP on 4T1 nodules in the lungs and establish preliminary dose-dependent effects, we performed a dose-finding study. Within the range of a single IV dose of DcNP carrying 0.125 to 50 mg/kg gemcitabine and 10/1 w/w paclitaxel (0.0125 to 5 mg/kg), we followed the growth (based in 4T1-luc bioluminescence intensity) of tumor nodules as therapeutic outcome measures. No clinical behavioral or hematological effects were notable for animals in all treatment groups for the 14-day study. However, a reproducible measure is the weight loss detected within 4–6 days after dosing (S1 Fig). Thus, we used this measure as a gross toxicity, likely similar to that observed with general GI toxicity associated in humans treated with gemcitabine [14, 15]. As shown in Fig 2A, dose-dependent measures of tumor nodule count and tumor intensity were noted, and the trend of these two measures was a similarly graded response. At 20 mg/kg gemcitabine plus 2 mg/kg paclitaxel in DcNP, these two measures exhibit a 90–95% inhibition of 4T1-luc tumor burden, indicating a smaller dose requirement for cancer clearance compared to initial data presented in Fig 1. The same level of clearance was not achievable for the CrEL drug combination, even with 5-fold higher doses (i.e., 80–90% inhibition at 100/10 mg/kg GT). The 50% effective doses (ED_50_s) for GT in DcNP fixed dose combinations were determined to be 1.655/0.1655 and 2.958/0.2958 mg/kg based on luminescence intensity and nodule count respectively (Fig 2B). Based on the 20% weight loss (a maximum number allowable for experimental study) as a gross toxicology measure, the dose-dependent weight loss profile exhibited a much higher dose range and did not occur until 30/3 GT mg/kg dose. The dose-response curve for weight loss, referred to as a toxic dose (TD) is steeper and well-separated from the GT DcNP dose range that inhibited 4T1-luc tumor. The 50% toxic or TD_50_ was determined to be 36.48/3.648 mg/kg GT. Using the mid-point of effective dose and toxic (weight-loss) doses, and the average therapeutic index, the ratio of toxicity-to-effective dose is estimated to be about 15.8 for GT DcNP. Taken together, dose-ranging studies indicate that the effective dose range is lower and well-separated from the toxicity range for GT combination by about 16 fold when given in DcNP dosage form. These data also confirmed that a single IV dose may clear a significant burden of invasive 4T1 with a sufficient margin of safety.

### Reproducibility of GT DcNP’s physicochemical and *in vivo* study data

Due to inherent challenges in translating nanomedicine into clinical applications, it is important to validate the reproducibility of our GT DcNP regarding physicochemical properties and *in vivo* effectiveness. Due to high variance of the animal models, we carried out at least two independent effectiveness studies for each DcNP dose reported in Fig 2. A two-way ANOVA analysis indicates that the difference between mean bioluminescence of replications for the same doses are insignificant but significant between difference doses (*p* = 0.0854 for replication factor and *p*<0.0001 for dose factor). Our results verified that the DcNPs produced in different batches exhibit similar characteristics—nearly identical mean particle size, drug loading and association efficiency, and more importantly, the ability to inhibit 4T1 metastasis in mice; thus these results were reproducible and consistent.

## Discussion

With early detection and targeted therapies, breast cancer survival rates have increased [1], even as a cure remains elusive. When dealing with advanced metastatic disease, highly potent but toxic combination regimens such as GT are a limited option with dose-limiting toxicity as a significant barrier [16]. Capitalizing on the drug combination nanoparticle (DcNP) platform’s ability to stabilize water soluble gemcitabine and insoluble paclitaxel together into GT DcNP, we found that the DcNP enhanced the plasma drug exposure at higher and longer lasting levels in mice. In a 4T1-luc metastatic mouse model, a single dose of GT DcNP was able to completely suppress 4T1 metastasis in the lung tissues. Dose-response studies also revealed the enhanced efficacy of GT drug combination and extended safety margin when given in GT DcNP dosage form.

For metastatic breast cancer treatment, combination therapy has been proven more effective than monotherapy [6]. However—due to the disparate physicochemical properties—co-formulation for the targeted delivery of hydrophilic and hydrophobic drugs such as GT has been challenging. The disparate physical properties of the two drug combinations prevent them from associating together in a stable form. While liposome encapsulation of doxorubicin—via a complex manufacturing process removing unbound drug and precise remote loading—is available as Doxil, it is a single agent that must be combined with other agents expressing different pharmacokinetics, tissue distribution, and time courses for combination therapy. The benefit of liposomal doxorubicin is derived from enhanced tumor tissue accumulation through the neovasculature, which is formed at a later stage of tumor nodule development. A product with two-drugs encapsulated in liposomes called Vyxeos was recently approved by the FDA. Vyxeos contains two water soluble drugs cytarabine and daunorubicin. However, both drugs were subjected to labor intensive purification mentioned above (followed by lyophilization as a finished product) and only intended for treating leukemia, which exhibits significantly different cancer biology from metastatic breast cancer. Unlike liposome encapsulation, the DcNP platform is based on water-soluble (i.e., gemcitabine) and insoluble (i.e. paclitaxel) co-solubilized in a soft organic solvent (i.e., ethanol) together with lipid excipients serving as a bridge or glue. Removal of the solvent and rehydration allows the formation of a stable drug-combination complex that can be broken down into GT DcNP particles at a size that is amenable for use as an injectable dosage form. Thus, this simplified process requires no unbound drug separation, purification, or lyophilization, which could help with product scaling, reproducibility and cost-saving.

The current human GT combination is given in a sequence with IV infusions of paclitaxel followed by gemcitabine (after 2–3 h) at doses of ~1250/175 mg/m^2^ GT, equivalent to ~35/3.5 mg/kg [6, 7]. Sequential dosing of conventional GT is necessary both to improve tolerability and reduce toxicity. In our study, we were able to combine both drugs while exhibiting sufficient safety in mouse models. The effective dose-range 10-50/1-5 mg/kg GT in mice is within the current range of human doses given in multiple cycles. While the exact mechanisms leading to this enhanced therapeutic index is not clear, it is likely through enhancement in differential drug distribution and pharmacokinetic profile. With two drugs in one intravenous injection, the DcNP formulation has prolonged the apparent elimination half-life of gemcitabine by more than 8× and enhanced its AUC by nearly 60×, higher than known previous achievements [17, 18]. Such enhancement may owe to association to DcNP, together with reduced paclitaxel clearance; plus potentially prevent exposure of DcNP bound gemcitabine inactivation by cytidine deaminase (to 2',2'-difluorodeoxyuridine, or dFdU) in liver and cells [19]. Regardless of pharmacokinetic and physiologic mechanisms, the DcNP formulation has enhanced the GT pharmacokinetic and pharmacodynamic profile resulting in an ~10-fold lower GT dose needed to inhibit metastatic cancer with a safety margin (TI of 15.8).

The therapeutic effects mediated by DcNP on GT were evaluated in 4T1 inoculated systematically to produce the lung metastasis model. This model is immunocompetent and relevant to human disease, where immune contribution is important. A genomic profiling study revealed a high consistency between lung metastases from orthotopic (mammary fat pad) and IV inoculation models, demonstrating that this approach mimics the spread of metastatic breast cancer cells from the primary tumor site [20]. This model is also clinically relevant to human disease due to reported spontaneous 4T1 metastasis to the lungs, brain, and bones in mice with functional immune systems [21]. Models generated with murine originated 4T1 cells have proven useful in metastasis disease and interventional studies [3, 4], and are used extensively in discovering immuno- and chemotherapeutics targeting metastatic breast cancer [22, 23].

Limited by safety concerns and the short half-lives of most current chemotherapeutic drugs, repeated dosing regimens are typically used in humans. Single agent drug resistance, and cumulative drug toxicity for some drugs could limit treatment options for metastatic diseases. In mouse model of this study, cancer cells progress into the lungs from the blood to cause mortality in only ~14 days. In this time window, the highest achievable single dose of the CrEL drug combination (limited by paclitaxel solubility) inhibited the process marginally. For 80~90% inhibition of lung metastasis of 4T1 within a similar timeframe, multiple dosing was required in previous studies [24–27]. Even with more sophisticated nanoparticles with target-activated drug delivery (e.g., legumain), none of the reports were able to demonstrate clearing of the 4T1 lung metastasis in a single dose [28]. In contrast, GT DcNP inhibited 100% of the lung metastasis with no detectable cancer in vivo and ex vivo with only one single IV injection (Figs 1 and 2). Whether a lower dose could be given in a multiple dosing scheme to produce a higher safety margin or TI is unclear but further study is warranted. It should be noted that the intent of this 4T1 mouse model is to allow the evaluation of drug-combinations to inhibit and clear the metastatic spread of advancing breast cancer cells in blood and for lung nodules. Thus, it is not intended to evaluate effects on primary breast cancer which may be laden with neovasulature amenable for nanoparticle mediated drug delivery through commonly prescribed enhanced permeability and retention or EPR effect. The enhanced 4T1 clearance of lung nodule formation is unlikely due to EPR. In addition, at a late metastatic stage (colonies established in lungs), whether a single or multiple GT DcNP dose in this present form would be effective remains a topic for additional investigations. Furthermore, GT ratio and excipient composition may also play a role in overall therapeutic and pharmacokinetic outcomes.

The ability of DcNP to enhance GT combination also opens other opportunities to enhance cancer chemotherapy. As lymphatic cancer invasion is believed to be an early site for metastasis, it is possible that DcNP loaded drugs may provide additional benefits. With at least 4 sets of HIV drug combinations formulated in DcNP, all drugs have been given subcutaneously in DcNP dosage for each composition into lymphatics in their first-pass [8–11, 29]. After subcutaneous administration and during the first passage, DcNP particles are shown to track preferentially into the lymph, but not blood capillaries, as observed with a near-infrared fluorophore (indocyanine green) tagged DcNP in mice [29]. In addition, DcNP has enabled the maintenance of cellular drug levels in lymph node mononuclear cells (above plasma drug levels) for over 2–4 weeks in non-human primates (NHP) [9, 10]. In our current report, we were able to demonstrate less dramatic long-acting effects due to DcNP for GT combinations when given in an IV dosage form. With an alternative subcutaneous route of GT DcNP administration, it is possible that drugs could be localized in the lymphatic system and stand ready to block the lymphatic metastasis pathway.

In summary, we have developed a simple, stable, and scalable GT DcNP dosage suitable for IV administration that transforms short-acting gemcitabine into a long-acting variation. We have also enhanced the pharmacokinetic profile of both drugs. The enhanced GT pharmacokinetic profile provided by DcNP dosage form parallels the GT effect against 4T1 metastatic breast cancer. A single GT DcNP injection completely inhibited lung metastasis in mice at levels that cannot be achieved with a CrEL dosage form at equal or higher doses. The enhanced dose-response was observed with a significant margin of safety. With the flexibility of the DcNP platform and impact on GT pharmacokinetics, pharmacodynamics, and safety windows, this approach could be generally applicable for use with other drug combinations intended to treat a number of cancer types, especially for the metastatic disease stage. The ability to transform current short-acting drugs into long-acting forms with preferential uptake could also accelerate clinical translation of the drug combinations in DcNP dosage form.

## Supporting information

S1 Fig Time course body weight changes in 4T1-inoculated mice treated with placebo (saline), GT in Cremophor EL/EtOH/PBS (CrEL) suspension or DcNP (drug-combination nanoparticle) dosage form. On day 0 GT in CrEL suspension or DcNP at 1.25/0.125, 10/1, or 50/5 mg/kg IV doses, and the 4T1 inoculated mice were monitored over 14 days. Each treatment group contains 8–15 mice and the data presented are mean ± SEM. In the group of 50/5 mg/kg of DcNP treated mice, some animals, due to clinical necessity, were sacrificed ahead of schedule. The remaining animals in the high dose group recovered and by day 14 appeared to exhibit body weight higher than at entry. In comparison, the saline placebo treated animals exhibit significant (15%) weight loss by day 14 due to rapid growth of lung metastatic nodules. The same trend is seen in the group treated with GT in CrEL suspensions (at two lower doses—10/1 and 1.25/0.125 mg/kg).
(TIF)

## References

[1] Siegel RL, Miller KD, Jemal A. Cancer statistics, 2019. CA-Cancer J Clin. 2019;69(1):7–34. 10.3322/caac.21551 WOS:000455538900003. 30620402

[2] Steeg PS. Tumor metastasis: mechanistic insights and clinical challenges. Nature Medicine. 2006;12:895 10.1038/nm1469 16892035

[3] Brown M, Assen FP, Leithner A, Abe J, Schachner H, Asfour G, et al Lymph node blood vessels provide exit routes for metastatic tumor cell dissemination in mice. Science. 2018;359(6382):1408–11. 10.1126/science.aal3662 29567714

[4] Pereira ER, Kedrin D, Seano G, Gautier O, Meijer EFJ, Jones D, et al Lymph node metastases can invade local blood vessels, exit the node, and colonize distant organs in mice. Science. 2018;359(6382):1403–7. Epub 2018/03/24. 10.1126/science.aal3622 29567713 PMC6002772

[5] NCCN Guidelines® 2019 [01/03/2020]. Available from: https://www.nccn.org/professionals/physician_gls/default.aspx.

[6] Albain KS, Nag SM, Calderillo-Ruiz G, Jordaan JP, Llombart AC, Pluzanska A, et al Gemcitabine plus Paclitaxel versus Paclitaxel monotherapy in patients with metastatic breast cancer and prior anthracycline treatment. Journal of clinical oncology: official journal of the American Society of Clinical Oncology. 2008;26(24):3950–7. Epub 2008/08/20. 10.1200/jco.2007.11.9362 . 18711184

[7] Xu B, Shen Z, Jiang Z, Guan Z, Zhang X. A phase II study of gemcitabine plus paclitaxel in patients with metastatic breast cancer and prior anthracycline treatment. Asia-Pacific journal of clinical oncology. 2010;6(4):320–9. Epub 2010/12/01. 10.1111/j.1743-7563.2010.01323.x . 21114782

[8] Koehn J, Iwamoto JF, Kraft JC, McConnachie LA, Collier AC, Ho RJY. Extended cell and plasma drug levels after one dose of a three-in-one nanosuspension containing lopinavir, efavirenz, and tenofovir in nonhuman primates. AIDS (London, England). 2018;32(17):2463–7. Epub 2018/08/14. 10.1097/qad.0000000000001969 . 30102655 PMC6482845

[9] Kraft JC, McConnachie LA, Koehn J, Kinman L, Collins C, Shen DD, et al Long-acting combination anti-HIV drug suspension enhances and sustains higher drug levels in lymph node cells than in blood cells and plasma. AIDS (London, England). 2017;31(6):765–70. Epub 2017/01/19. 10.1097/qad.0000000000001405 28099191 PMC5345888

[10] McConnachie LA, Kinman LM, Koehn J, Kraft JC, Lane S, Lee W, et al Long-Acting Profile of 4 Drugs in 1 Anti-HIV Nanosuspension in Nonhuman Primates for 5 Weeks After a Single Subcutaneous Injection. Journal of pharmaceutical sciences. 2018;107(7):1787–90. Epub 2018/03/20. 10.1016/j.xphs.2018.03.005 . 29548975 PMC6954863

[11] Perazzolo S, Shireman LM, Koehn J, McConnachie LA, Kraft JC, Shen DD, et al Three HIV Drugs, Atazanavir, Ritonavir, and Tenofovir, Coformulated in Drug-Combination Nanoparticles Exhibit Long-Acting and Lymphocyte-Targeting Properties in Nonhuman Primates. Journal of pharmaceutical sciences. 2018;107(12):3153–62. Epub 2018/08/20. 10.1016/j.xphs.2018.07.032 30121315 PMC6553477

[12] Srivastava S, Riddell S. Adoptive therapy of ROR1+ murine solid tumors with chimeric antigen receptor-modified T cells (VAC7P.1045). The Journal of Immunology. 2015;194(1 Supplement):143.15–.15.

[13] Tao K, Fang M, Alroy J, Sahagian GG. Imagable 4T1 model for the study of late stage breast cancer. BMC Cancer. 2008;8(1):1–19. 10.1186/1471-2407-8-228 18691423 PMC2529338

[14] Beane JD, Griffin KF, Levy EB, Pandalai P, Wood B, Abi-Jaoudeh N, et al Duodenal ischemia and upper GI bleeding are dose-limiting toxicities of 24-h continuous intra-arterial pancreatic perfusion of gemcitabine following vascular isolation of the pancreatic head: early results from the Regional Chemotherapy in Locally Advanced Pancreatic Cancer (RECLAP) study. Investigational New Drugs. 2015;33(1):109–18. 10.1007/s10637-014-0157-7 25236592 PMC5542811

[15] Loehrer PJ Sr., Feng Y, Cardenes H, Wagner L, Brell JM, Cella D, et al Gemcitabine alone versus gemcitabine plus radiotherapy in patients with locally advanced pancreatic cancer: an Eastern Cooperative Oncology Group trial. Journal of clinical oncology: official journal of the American Society of Clinical Oncology. 2011;29(31):4105–12. Epub 10/03. 10.1200/JCO.2011.34.8904 . 21969502 PMC3525836

[16] Lim B, Hortobagyi GN. Current challenges of metastatic breast cancer. Cancer and Metastasis Reviews. 2016;35(4):495–514. 10.1007/s10555-016-9636-y 27933405

[17] Paolino D, Cosco D, Racanicchi L, Trapasso E, Celia C, Iannone M, et al Gemcitabine-loaded PEGylated unilamellar liposomes vs GEMZAR®: Biodistribution, pharmacokinetic features and in vivo antitumor activity. Journal of Controlled Release. 2010;144(2):144–50. 10.1016/j.jconrel.2010.02.021 20184929

[18] Zhang J, Zhang P, Zou Q, Li X, Fu J, Luo Y, et al Co-Delivery of Gemcitabine and Paclitaxel in cRGD-Modified Long Circulating Nanoparticles with Asymmetric Lipid Layers for Breast Cancer Treatment. Molecules. 2018;23(11):2906 10.3390/molecules23112906 . 30405089 PMC6278289

[19] Frese KK, Neesse A, Cook N, Bapiro TE, Lolkema MP, Jodrell DI, et al nab-Paclitaxel potentiates gemcitabine activity by reducing cytidine deaminase levels in a mouse model of pancreatic cancer. Cancer Discov. 2012;2(3):260–9. Epub 02/28. 10.1158/2159-8290.CD-11-0242 . 22585996 PMC4866937

[20] Rashid OM, Nagahashi M, Ramachandran S, Dumur CI, Schaum JC, Yamada A, et al Is tail vein injection a relevant breast cancer lung metastasis model? Journal of thoracic disease. 2013;5(4):385–92. Epub 2013/08/31. 10.3978/j.issn.2072-1439.2013.06.17 23991292 PMC3755653

[21] Pulaski BA, Ostrand-Rosenberg S. Mouse 4T1 Breast Tumor Model. Current Protocols in Immunology. 2000;39(1):20.2.1–.2.16. 10.1002/0471142735.im2002s39 18432775

[22] Eckhardt BL, Francis PA, Parker BS, Anderson RL. Strategies for the discovery and development of therapies for metastatic breast cancer. Nature Reviews Drug Discovery. 2012;11:479 10.1038/nrd2372 https://www.nature.com/articles/nrd2372#supplementary-information. 22653217

[23] Rashid OM, Takabe K. Animal models for exploring the pharmacokinetics of breast cancer therapies. Expert Opin Drug Metab Toxicol. 2015;11(2):221–30. Epub 11/21. 10.1517/17425255.2015.983073 . 25416501 PMC4583421

[24] Cao H, Zhang Z, Zhao S, He X, Yu H, Yin Q, et al Hydrophobic interaction mediating self-assembled nanoparticles of succinobucol suppress lung metastasis of breast cancer by inhibition of VCAM-1 expression. Journal of Controlled Release. 2015;205:162–71. 10.1016/j.jconrel.2015.01.015 25598420

[25] Cao H, Dan Z, He X, Zhang Z, Yu H, Yin Q, et al Liposomes Coated with Isolated Macrophage Membrane Can Target Lung Metastasis of Breast Cancer. ACS Nano. 2016;10(8):7738–48. 10.1021/acsnano.6b03148 27454827

[26] Dan Z, Cao H, He X, Zhang Z, Zou L, Zeng L, et al A pH-Responsive Host-guest Nanosystem Loading Succinobucol Suppresses Lung Metastasis of Breast Cancer. Theranostics. 2016;6(3):435–45. 10.7150/thno.13896 . 26909117 PMC4737729

[27] Chen Q, Ross AC. All-trans-retinoic acid and the glycolipid α-galactosylceramide combined reduce breast tumor growth and lung metastasis in a 4T1 murine breast tumor model. Nutr Cancer. 2012;64(8):1219–27. 10.1080/01635581.2012.718404 . 23163850 PMC5460978

[28] He X, Cao H, Wang H, Tan T, Yu H, Zhang P, et al Inflammatory Monocytes Loading Protease-Sensitive Nanoparticles Enable Lung Metastasis Targeting and Intelligent Drug Release for Anti-Metastasis Therapy. Nano Letters. 2017;17(9):5546–54. 10.1021/acs.nanolett.7b02330 28758755

[29] Kraft JC, Treuting PM, Ho RJY. Indocyanine green nanoparticles undergo selective lymphatic uptake, distribution and retention and enable detailed mapping of lymph vessels, nodes and abnormalities. Journal of drug targeting. 2018;26(5–6):494–504. Epub 2018/02/02. 10.1080/1061186X.2018.1433681 29388438 PMC6205717

