# Supplementary figures and images for: Novel drug combination nanoparticles exhibit enhanced plasma exposure and dose-responsive effects on eliminating breast cancer lung metastasis
Source: PLoS One. 2020 Mar 6;15(3):e0228557. doi: 10.1371/journal.pone.0228557 (PMC7059902; doi:10.1371/journal.pone.0228557)

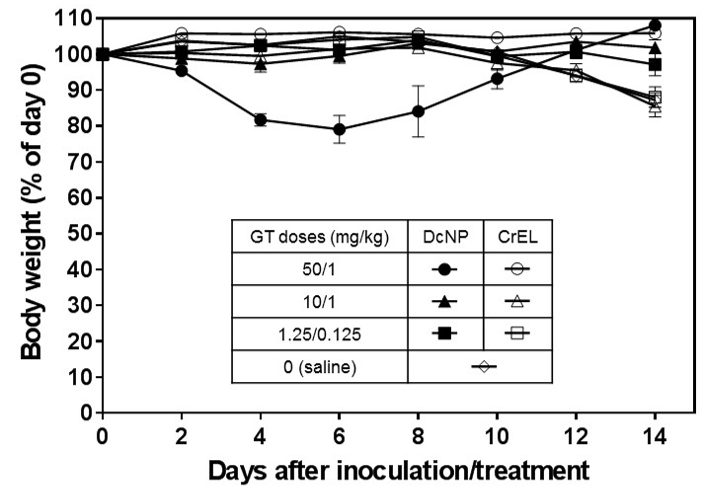

Supplement: S1 Fig — On day 0 GT in CrEL suspension or DcNP at 1.25/0.125, 10/1, or 50/5 mg/kg IV doses, and the 4T1 inoculated mice were monitored over 14 days. Each treatment group contains 8–15 mice and the data presented are mean ± SEM. In the group of 50/5 mg/kg of DcNP treated mice, some animals, due to clinical necessity, were sacrificed ahead of schedule. The remaining animals in the high dose group recovered and by day 14 appeared to exhibit body weight higher than at entry. In comparison, the saline placebo treated animals exhibit significant (15%) weight loss by day 14 due to rapid growth of lung metastatic nodules. The same trend is seen in the group treated with GT in CrEL suspensions (at two lower doses—10/1 and 1.25/0.125 mg/kg). (TIF) [file pone.0228557.s001.tif]
